# Antitumoral Activity of the MEK Inhibitor Trametinib (TMT212) Alone and in Combination with the CDK4/6 Inhibitor Ribociclib (LEE011) in Neuroendocrine Tumor Cells In Vitro

**DOI:** 10.3390/cancers13061485

**Published:** 2021-03-23

**Authors:** Xi-Feng Jin, Gerald Spöttl, Julian Maurer, Svenja Nölting, Christoph Josef Auernhammer

**Affiliations:** 1 Medizinische Klinik 4, LMU Klinikum, Klinikum der Universitaet Muenchen, Ludwig-Maximilians-University of Munich, Campus Grosshadern, Marchioninistrasse 15, 81377 Munich, Germany; xifeng.jin@campus.lmu.de (X.-F.J.); gerald.spoettl@med.uni-muenchen.de (G.S.); Julian.Maurer@med.uni-muenchen.de (J.M.); Svenja.Noelting@med.uni-muenchen.de (S.N.); 2 Interdisciplinary Center of Neuroendocrine Tumors of the GastroEnteroPancreatic System (GEPNET-KUM), LMU Klinikum, Klinikum der Universitaet Muenchen, Ludwig-Maximilians-University of Munich, Campus Grosshadern, Marchioninistrasse 15, 81377 Munich, Germany; 3 Klinik für Endokrinologie, Diabetologie und Klinische Ernährung, Universitätsspital Zürich, Raemistrasse 100, CH-8091 Zürich, Switzerland

**Keywords:** neuroendocrine tumor, MEK, ERK, CDK4/6

## Abstract

**Simple Summary:**

Systemic treatment options for advanced neuroendocrine tumors have significantly been improved in the last decade. However efficacy of systemic therapy is limited by tumor resistance and therefore there is a need for further treatment options. Inhibition of the Ras-Raf-Mek-Erk signaling cascade might be a promising new treatment strategy in neuroendocrine neoplasms. In this study we investigated the effects of the MEK inhibitor trametinib, the ERK inhibitor SCH772984 and the CDK4/6 inhibitor ribociclib in human neuroendocrine tumor cell lines BON1, QGP1 and NCI-H727 in vitro. Trametinib alone and in synergism with ribociclib demonstrated antiproliferative effects. Combination therapy of MEK inhibitors and CDK4/6 inhibitors might be a potential strategy to overcome CDK4/6 inhibitor resistance in neuroendocrine tumors.

**Abstract:**

Objectives: This study assessed the antitumoral activity of the MEK inhibitor trametinib (TMT212) and the ERK1/2 inhibitor SCH772984, alone and in combination with the CDK4/6 inhibitor ribociclib (LEE011) in human neuroendocrine tumor (NET) cell lines in vitro. Methods: Human NET cell lines BON1, QGP-1, and NCI-H727 were treated with trametinib or SCH772984, alone and in combination with ribociclib, to assess cell proliferation, cell cycle distribution, and protein signaling using cell proliferation, flow cytometry, and Western blot assays, respectively. Results: Trametinib and SCH772984, alone and in combination with ribociclib, significantly reduced NET cell viability and arrested NET cells at the G1 phase of the cell cycle in all three cell lines tested. In addition, trametinib also caused subG1 events and apoptotic PARP cleavage in QGP1 and NCI-H727 cells. A western blot analysis demonstrated the use of trametinib alone and trametinib in combination with ribociclib to decrease the expression of pERK, cMyc, Chk1, pChk2, pCDK1, CyclinD1, and c-myc in a time-dependent manner in NCI-H727 and QGP-1 cells. Conclusions: MEK and ERK inhibition causes antiproliferative effects in human NET cell lines in vitro. The combination of the MEK inhibitor trametinib (TMT212) with the CDK4/6 inhibitor ribociclib (LEE011) causes additive antiproliferative effects. Future preclinical and clinical studies of MEK inhibition in NETs should be performed.

## 1. Introduction

Neuroendocrine neoplasms (NENs) are a heterogeneous group of neuroendocrine tumors (NETs) and neuroendocrine carcinomas (NECs) that can occur in most parts of the human body, most often in the intestine, pancreas, and lungs [1,2]. Treatment of advanced neuroendocrine neoplasms is dependent on tumor classification, tumor grade, and localization of the primary tumor [3,4,5,6,7]. Current systemic treatment options for NETs include somatostatin receptor (ssr) expression-based biotherapy with somatostatin analogs, and peptide receptor radionuclide therapy (PRRT) with 177Lutetium_DOTA-TATE [8]. Further systemic treatment options include streptozotocin-based and temozolomide-based chemotherapy regimens in combination with 5-fluoropyrimidin analogues, as well as targeted therapy with the mTOR inhibitor everolimus or with several multi-tyrosine kinase inhibitors such as sunitinib, surufatinib, cabozantinib, lenvatinib, pazopanib, and others [8].

Although over the past decade, NET treatment strategies and treatment outcomes have improved [1], their efficacy is limited by resistance mechanisms, and further novel treatment strategies are urgently needed. 

During human carcinogenesis and cancer progression, tumor cells possess at least six essential hallmarks, i.e., tumor cell self-sufficiency in growth signals, insensitivity to growth–inhibitory signals, evasion of apoptosis, limitless replicative potential, sustained angiogenesis, and tissue invasion and metastasis [9]. 

NENs of the pancreas, small intestines, and lungs harbor various somatic mutations, as has been recently described [10,11,12]. For example, the proto-oncogenes K-RAS and BRAF, the mitogen-activated protein kinase (MAPK), the extra-cellular signal-regulated kinase (ERK), the phosphoinositide 3-kinase (PI3K)-PTEN-AKT, and CDK4/6-Rb signaling play essential roles in cancer initiation, promotion, and progression [10,11,12]. Mutations of genes, such as *KRAS*, *BRAF*, *PI3KCA*, *PTEN*, *CDKN2A*, *CDKN2B*, or *AKT*, can be found in NENs [10,11,12]. 

The CDK4/6 inhibitors ribociclib, palbociclib, and abemaciclib have been approved in the treatment of breast cancer in combination with endocrine therapy [13]. Abemaciclib has also been approved as a single agent in the treatment of breast cancer. CDK4/6 inhibitors (CDK4/6i) possess effective antitumor activity in a variety of solid tumors and are being currently investigated to treat various solid tumors in different phases I, II, and III in clinical trials [14]. 

In neuroendocrine tumors, the CDK4/6 inhibitor palbociclib (PD-0332991) demonstrated significant antitumoral activity in a preclinical in vitro and in vivo model [15]. By contrast, in a clinical phase II trial (NCT02806648) in *n* = 20 patients with pancreatic NETs, palbociclib failed to demonstrate clinically significant antitumoral effects, with best-response stable disease and a median progression-free survival of 2.6 months [16]. Recently, we demonstrated the CDK4/6 inhibitor ribociclib (LE011) to exhibit significant antitumoral activity in human neuroendocrine tumor cell lines in vitro [17]. In a clinical phase II trial (NCT02420691) in *n* = 20 patients with foregut NETs, ribociclib caused best-response stable disease and a median progression-free survival of 10.4 months [18].

CDK4/6 inhibitor resistance [19] might be overcome by additional MEK inhibition [20], and several clinical trials of CDK4/6 inhibitors such as ribociclib or palbociclib with MEK inhibitors such as trametinib and bimetinib are currently ongoing in various cancer models [20]. Checkpoint inhibitor therapy in neuroendocrine tumors has so far been mostly disappointing [21]. On the other hand, CDK4/6 inhibitors alone and in combination have also been reported to influence the tumor microenvironment and exert immunogenic effects [20]. Therefore, instead of a single drug treatment, the use of CDK4/6 inhibitors in combination treatment strategies could be an option to redevelop CDK4/6 inhibitors as an antitumoral strategy in NETs. MAPKs and CDKs can form complex kinase networks to interact and regulate cell survival and death [22], the targeting of which might provide a novel strategy for cancer therapy via enhancement of CDK4/6i activity and reduction of tumor resistance. Indeed, previous studies showed the synergistic antitumoral effects of a combination of CDK4/6 inhibitors and MEK inhibitors in neuroblastoma [23], colorectal cancer [24,25], non-small cell lung cancer [26], and melanoma [27,28]. Current clinical phase I trials in patients with advanced solid tumors have evaluated the combination therapy of the MEK inhibitor trametinib in combination with the CDK4/6 inhibitors palbociclib (NCT02065063) [29] and ribociclib (NCT0270351), respectively. 

The MEK inhibitor trametinib (TMT212), a selective allosteric inhibitor of MEK1/2, is able to control cancer cells with an overactive MEK-ERK pathway [30]. For the treatment of BRAF-mutated melanoma, various combination therapy regimens of a BRAF inhibitor plus MEK inhibitor, such as vemurafenib plus cobimetinib, encorafenib plus binimetinib, and dabrafenib plus trametinib, have been approved [31]. The latter combination of dabrafenib plus trametinib has also recently been approved for the treatment of BRAF-mutated non-small cell lung cancer (NSCLC) [32] and for the treatment of BRAF-mutated anaplastic thyroid carcinoma [33].

In neuroendocrine tumor cell lines, different MEK inhibitors have been reported to demonstrate antitumoral effects in vitro [34,35,36,37], as has been reported for PD0325901 [35], U0126 [36], and trametinib [37]. Trametinib has been reported to be more effective in NETs of pancreatic origin than in NETs of small intestinal origin, as demonstrated by in vitro experiments in permanent cell lines, as well as in human primary cultures of pancreatic and small intestinal NETs [37]. The human pancreatic NET cell line BON1 harbors a mutation in NRAS, while the human pancreatic NET cell line QGP-1 harbors a mutation in KRAS [38].

In this study, we further assess the effects of the MEK inhibitor (MEKi) trametinib (TMT212), the ERK1/2 inhibitor (ERKi) SCH772984, and their combination with the CDK4/6 inhibitor (CDK4/6i) ribociclib (LEE011) on NETs in vitro and explore the underlying molecular events. We provide insightful information regarding antitumoral efficacy of MEK inhibition alone and in combination with CDK4/6 inhibition in NETs. 

## 2. Materials and Methods

### 2.1. Cell Lines, Culture, Reagents, and Treatment

Human pancreatic NET BON1 cell line [37,38,39] was kindly gifted by Prof. R Goeke (University of Marburg, Marburg, Germany), while the pancreatic islet tumor cell line QGP-1 [37,38,39] was obtained from The Japanese Collection of Research Bioresources Cell Bank (Japan), both of which were maintained in Dulbecco’s modified Eagle’s medium (DMEM)/F12. The human bronchopulmonary neuroendocrine cell line NCI-H727 [37,39] was purchased from American Type Culture Collection (Manassas, VA, USA) and cultured in Roswell Park Memorial Institute medium-1640 (RPMI-1640). The growth media were supplemented with 10% fetal bovine serum (FBS), 1% penicillin/streptomycin, and 0.4% amphotericin B, and the cells were cultured in a humidified incubator with 5% CO_2_ at 37 °C. 

Ribociclib (LEE011) and trametinib (TMT212) were provided by Novartis (Basel, Switzerland), while SCH772984 was purchased from Selleckchem (Germany). These reagents were dissolved in dimethyl sulfoxide (DMSO) as stock solutions and stored at –20 °C. Their working solutions were used to treat NETs cells as single therapies and as combinations for different durations of time for various assays. 

### 2.2. Cell Proliferation Assays

To assess the effects of ribociclib (LEE011), trametinib (TMT212), and SCH772984 alone or in appropriate combinations, we utilized the Cell Titer Blue^®^ cell viability assay kit from Promega (Madison, WI, USA). In brief, NET cells were seeded into 96-well plates, grown overnight, and then treated with different doses of ribociclib (LEE011), trametinib (TMT212), SCH772984, and or their combinations for up to 144 h. Our group previously calculated the population doubling time (PDT) to be 0.895 ± 0.066 d for BON1, 1.536 ± 0.051 d for QGP-1, and 1.781 ± 0.295 d for NCI-H727 cells, respectively [40]. In addition, we have previously reported the effects of ribociclib (LEE011) in neuroendocrine tumor cells for up to 144 h [17]. Therefore, the different time points for cell proliferation for up to 144 h were used in the current experiments. The reference concentration of LEE011 was based on our previously published results [17].

At the end of each experiment, the cells were subjected to the kit procedures according to the manufacturer’s protocol. The optical density of each cell line was measured using a spectrophotometer (Promega, Madison, WI, USA) and inhibitory concentration IC_50_ values were calculated for each drug and cell line. 

### 2.3. Flow Cytometric Cell Cycle Distribution Assay

NET cells were seeded and grown in 6-well plates overnight and then treated with various doses of trametinib (TMT212), SCH772984, or trametinib (TMT212) plus ribociclib (LEE011) or SCH772984 plus ribociclib (LEE011) for 72 h. Next, the cells were harvested using 0.05% trypsin, centrifuged and washed in phosphate-buffered saline (PBS), then resuspended in the propidium iodide solution (Sigma-Aldrich, Taufkirchen, Germany) for flow cytometric analysis and quantification of the cell cycle distribution with BD Accuri C6 Analysis software (Biosciences, Heidelberg, Germany).

### 2.4. Western Blot

After NET cells were grown and treated with ribociclib (LEE011), trametinib (TMT212), or SCH772984, alone or in appropriate combinations, the total cellular protein was lysed in a lysis buffer containing the M-PER (Mammalian Protein Extraction Reagent) and the HALT protease and phosphatase inhibitor cocktail (Thermo Scientific, Rockford, IL, USA). The supernatants of the cell lysis were then quantified using the RotiQuant Universal protein assay kit (Roth, Karlsruhe, Germany). The protein samples containing 50 µg of each were separated in sodium dodecyl sulfate–polyacrylamide gel electrophoresis (SDS-PAGE) gels and transferred onto the polyvinylidene fluoride membranes (Millipore, Billerica, MA, USA). For Western blotting, the membranes were blocked in the Clear Milk Blocking Buffer (Pierce, Rockford, IL, USA) for 30 min and incubated at 4 °C overnight with different primary antibodies. The primary antibodies were anti-CyclinD1, CyclinD3, CyclinB1, CDK1, CDK4, CDK6, Chk1, pChk2 T68, Chk2, c-Myc, pEGFRY1068, EGFR, pIGF1RY1135, IGF1R, pAktS473, Akt, pp70S6K T389, p70S6K, p4EBP1 S65, 4EBP1, pERK T202/Y204, ERK, pMEK S217/221, MEK, pRB S780, pRSK1 S380, RSK, pJNK T183/Y185, JNK, and pp38 T180/Y182, p38, Puma, Mcl-1, Bcl-2, BclxL, Caspase 3, cleaved Caspase 3, PARP, pS6 S240/4,S6 (all from Cell Signaling Technology, Danvers, MA, USA), p21 Waf1/Cip1 (BD Transduction Laboratories, Franklin Lakes, NJ, USA), RB (Biolegend, San Diego, CA, USA), p53, AIF, and ß-Actin (Santa Cruz, Dallas, TX, USA).

On the next day, the membranes were washed with Tris-based saline-Tween 20 (TBS-T) briefly three times and then incubated with a horseradish-peroxidase-conjugated secondary antibody (Cell Signaling Technology, MA, USA) at a dilution of 1:25,000 for 2 h. After washing, the positive-bound protein bands were visualized with a chemiluminescence Western blotting detection system (WESTAR Supernova, Italy) and detected with an ECL imaging system (INTAS, Göttingen, Germany). The level of each protein sample was quantified using the ImageJ software (National Institute of Health, Bethesda, MD, USA).

### 2.5. Statistical Analysis

All experiments were performed in triplicate and repeated at least once. The data were summarized as the mean ± SD of the triplicate samples and statistically analyzed using a one-way analysis of variance test (ANOVA) followed by Dunnett’s t test for multiple comparisons. Biosoft CalcuSyn 2.1 software (Ferguson, MO, USA) was used to assess the synergistic effects of the combination treatments. Here, *p* < 0.05 was considered statistically significant.

## 3. Results

### 3.1. The MEKi Trametinib and the ERKi SCH772984 Suppress NET Cell Proliferation 

We have previously reported effects of ribociclib (LEE011) in neuroendocrine tumor cells for up to 144 h [17]. The IC_50_ for ribociclib (LEE011) has been previously reported by our group [17] as follows: BON1 cells IC_50_ = 2.6 μM, QGp-1 cells IC_50_ = 1.2 μM, H727 cells IC_50_ = 10.9 μM.

**Figure 1:**
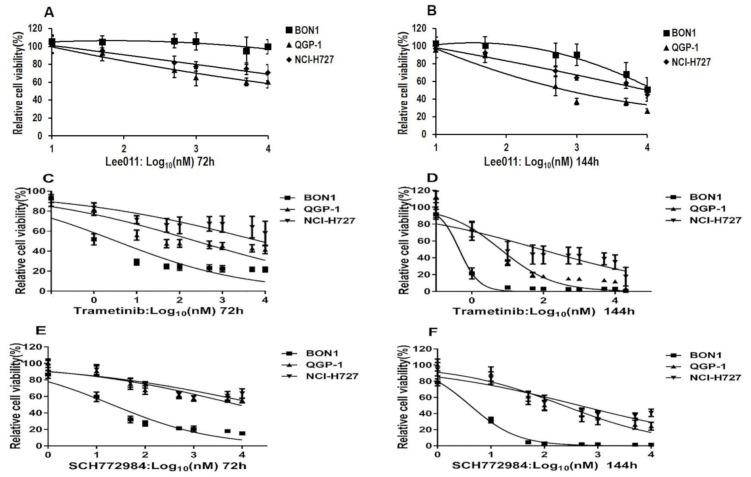
Effects of ribociclib (LEE011), trametinib or SCH772984 on reduction of neuroendocrine tumor (NET) cell viability. NET BON1, QGP-1, and NCI-H727 cell lines were grown and treated with various doses of ribociclib (LEE011) (**A**,**B**), trametinib (**C**,**D**) and SCH772984 (**E**,**F**) for 72 h and 144 h, respectively, and then subjected to a Cell Titer Blue^®^ cell viability assay. The log-transformed concentration values and the effect data were fitted to a four-parameter logistic equation.

In this study, we first assessed the effects of ribociclib (LEE011), trametinib (TMT212), and SCH772984 on regulation of NET cell proliferation when used in a panel of NET cell lines (Figure 1 logarithmic data and Figure S1 linear data). The NET cells were treated with varying doses of ribociclib (LEE011) (0–25 µM), trametinib (0–10 µM) and SCH772984 (0–20 µM) for 72 and 144 h, respectively (Figure 1 logarithmic data and Figure S1 linear data). Our data showed that after the 144 h treatment with the highest concentration, the median cell viability values after the trametinib treatment were 0.7%, 3.77%, and 17.58% for BON1, QGP-1, and NCI-H727 cells, respectively. The trametinib IC_50_ values were 0.44 nM, 6.359 nM, and 84.12 nM for BON1, QGP-1, and NCI-H727 cells, respectively (Figure 1). The median cell viability values after the SCH772984 treatment were 1.46%, 24.39%, and 40.82% for BON1, QGP-1, and NCI-H727, respectively. The SCH772984 IC_50_ values were 4.1 nM, 228 nM, and 454.5 nM for BON1, QGP-1, and NCI-H727, respectively (Figure 1). These data indicate that all of these three NET cell lines exhibited sensitivity to MEKi trametinib or ERKi SCH772984 treatment, and the pancreatic NET cell lines BON1 and QGP-1 were more sensitive than the lung NET cell line NCI-H727.

### 3.2. Synergistic Inhibitory Effects of the MEKi Trametinib (TMT212) or the ERKi SCH772984 in Combination with the CDK4/6i Ribociclib (LEE011) on NET Cell Proliferation

**Figure 2:**
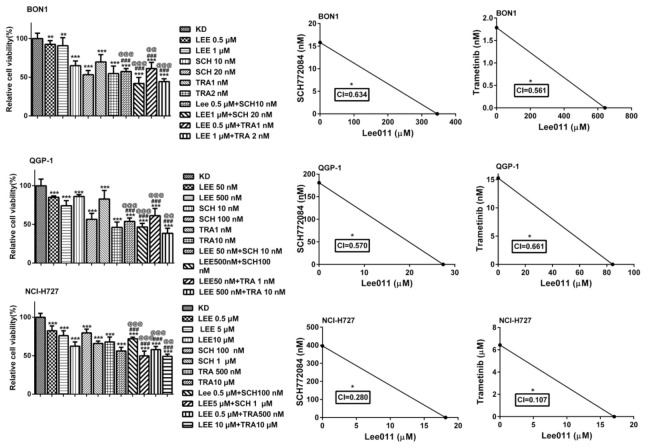
Effects of trametinib, SCH772984, and LEE011 in single treatments or combinations on the inhibition of NET cell viability. Various NET cell lines (BON1, QGP-1, and NCI-H727) were grown and treated with trametinib, SCH772984, and LEE011 in single treatments or combinations for 72h and 144 h and then subjected to the Cell Titer Blue^®^ cell viability assay kit. Note: * *p* < 0.05, ** *p* < 0.01, and *** *p* < 0.001 vs. the controls; ^#^ *p* <0.05, ^##^ *p* <0.01 and ^###^ *p* < 0.001 vs. LEE011 alone; ^@^ *p* < 0.05, ^@@^ *p* < 0.01, and ^@@@^ *p* < 0.001 vs. trametinib or SCH772984 alone.

We then examined the effects of the MEKi trametinib or the ERKi SCH772984 in combination with the CDK4/6i LEE011 on NET cell proliferation (Figure 2 and Figure S2). After treatment of the BON1, QGP-1, and NCI-H727 cell lines with single and combined drugs in various doses and different durations of time, we calculated the combination index (CI) values and the Fraction affected (Fa) of each dose of these reagents and used these values to generate the Fa–CI plots using CompuSyn software [41,42]. We defined CI < 1 as synergism and found that the computed CI values of the combination of LEE011 with trametinib were 0.561, 0.661, and 0.107 in BON1, QGP-1, and NCI-H727, respectively, while the CI values of the combination of LEE011 with SCH772984 were 0.634, 0.570 and 0.280, respectively (Figure 2). These data suggest that the combination of trametinib or SCH772984 with LEE011 had a synergistic effect on the inhibition of NET cell viability.

### 3.3. Synergistic Effects of the MEKi Trametinib (TMT212) or the ERKi SCH772984 in Combination with the CDK4/6i Ribociclib (LEE011) on NET Cell Cycle G1 Arrest, Sub G1 Events and Apoptosis

**Figure 3:**
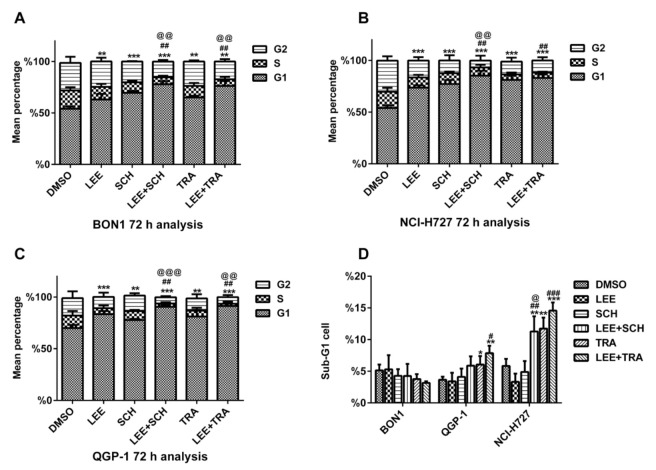
Effects of trametinib, SCH772984, and LEE011 in single treatments or combinations on NET cell G1 phase arrest (**A**–**C**) and increase in the subG1 cell population (**D**). Different NET cell lines (BON1, QGP-1, and NCI-H727) were grown and treated with trametinib (1–500 nm), SCH772984 (10–100 nM), and LEE011 (500nM) in single treatments or combinations for 72 h and assayed for cell cycle distribution using the flow cytometric assay. Note: * *p* < 0.05, ** *p* < 0.01, *** *p* < 0.001 vs. the controls; ^#^ *p* <0.05, ^##^ *p* <0.01, ^###^ *p* < 0.001 vs. LEE011 alone; ^@^ *p* < 0.05, ^@@^ *p* < 0.01, ^@@@^ *p* < 0.001 vs. trametinib or SCH772984 alone.

Subsequently, we examined the effects of the MEKi trametinib or the ERKi SCH772984 in combination with the CDK4/6i LEE011 on NET cell cycle distributions using flow cytometry analysis (Figure 3). A 72 h treatment with trametinib and SCH772984 alone arrested tumor cells in the G1 phase of the cell cycle (65.13% and 69.65% vs. 54.16% in the untreated control in BON1; 81.11% and 78.89% vs. 69.01% in the untreated control in QGP-1 cells; and 81.21% and 77.21% vs. 54% in the untreated control in NCI-H727, respectively). The combination of trametinib with LEE011 versus trametinib alone caused a significantly (*p* < 0.05) stronger cell cycle G1 arrest in BON1 cells (76.31% vs. 65.13%, respectively) and in QGP-1 cells (91.38% vs. 81.11%, respectively), and a corresponding decrease in cells in S and G2-M phases. Similarly, the combination of SCH772984 with LEE011 versus SCH772984 alone caused a significantly (*p* < 0.05) stronger cell cycle G1 arrest in BON1 cells (77.93% vs. 69.65%, respectively), QGP-1 cells (90.5% vs. 78.89%, respectively), and NCH-H727 cells (85.36% vs. 77.21%, respectively), and a corresponding decrease in cells in the S-phase in all three cell lines (Figure 3).

**Figure 4:**
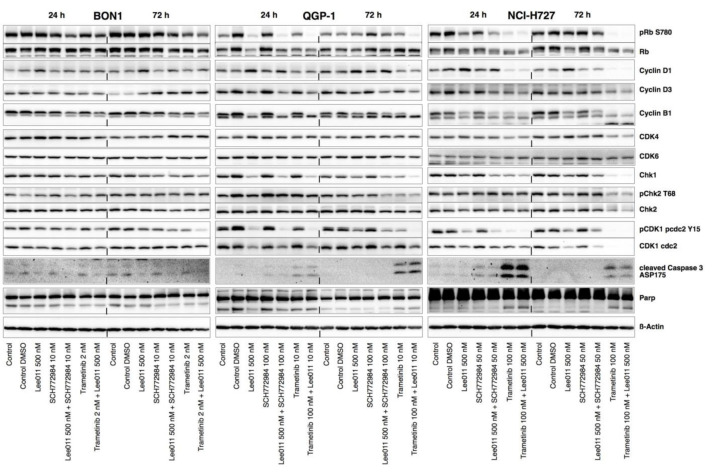
Effects of trametinib, SCH772984, and LEE011 in single treatments or combinations on regulation of CDK4/6-Rb signaling, apoptosis, and cell-cycle-related protein in NET cells. These NET cell lines (BON1, QGP-1, and NCI-H727) were grown and treated with trametinib, SCH772984, and LEE011 in single treatments or combinations for 24 or 72 h and analyzed using Western blotting for expression of CDK4/6, Rb, and apoptosis and cell-cycle-related proteins. Equal protein loading was verified by normalization to the total protein staining and by the housekeeping protein β-actin. Corresponding quantitative densitometry data are shown in Figure S4A–C.

Trametinib alone, as well as the combination of trametinib with LEE011, caused an increase in the subG1 cell population in QGP-1 and NCI-H727 cells, respectively (Figure 3D). A moderate induction of cell apoptosis in QGP-1 and NCI-H727 cells, was demonstrated by a moderate increase in cleaved caspase 3 and cleaved PARP (Figure 4, Figures S3 and S4C).

### 3.4. Synergistic Effects of the MEKi Trametinib (TMT212), the ERKi SCH772984, and the CDK4/6i Ribociclib (LEE011) on CDK4/6/Rb Signaling in NET Cell Lines

LEE011 alone, trametinib plus LEE011, and SCH772984 plus LEE011 exhibited significant reductions in pRb levels at 24 and at 72 h in QGP-1 and in NCI-H727 cells (Figure 4, Figure S4A). It is worth mentioning that trametinib plus LEE011 almost completely diminished the expression of pRb in QGP-1 cells and in NCI-H727 cells.

**Figure 5:**
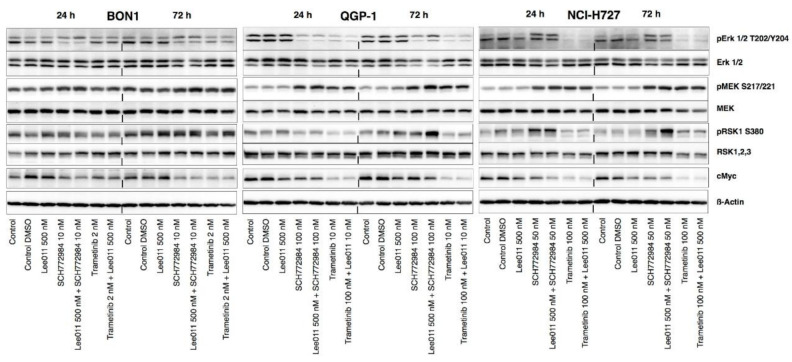
Effects of trametinib, SCH772984, and LEE011 in single treatments or combinations on the regulation of MEK/ERK signaling in NETs. NET cell lines (BON1, QGP-1, and NCI-H727) were grown and treated with trametinib, SCH772984, and LEE011 in single treatments or combinations for 24 or 72 h, and assayed using Western blotting to assess levels of the MEK/ERK signaling proteins. Corresponding quantitative densitometry data are shown in Figure S5.

Furthermore, trametinib in combination with LEE011 also sharply reduced the expression of Chk1, pChk2, CDK1, pCDK1, CDK4, and CDK6, as well as cyclin-dependent kinases (cyclinD1/D3, cyclin B1) (Figure 4B,C and Figure S4A), and c-Myc (Figure 5 and Figure S5), in a time-dependent manner in QGP-1 and NCI-H727 cells.

However, LEE011 combined with SCH772984 maintained the high levels of CDK4/6 and slightly decreased cyclinD1/D3 levels (Figure 4 and Figure S4A,B). The time course data also showed an initial decrease in CDK4, CDK1, and pCDK1 at 24 h, but a rebound of CDK4, CDK1, and pCDK1 levels over time in all three cell lines.

Thus, the MEK and ERK inhibitors induced the effects of LEE011 on NET cells, suggesting that the inhibition of the MAPK signaling could at least partially confer the sensitivity of tumor cells to LEE011.

### 3.5. Synergistic Downregulation of the MEK/ERK and MYC Signaling after Treatment of NET Cells with Ribociclib (LEE011) and Trametinib or Ribociclib (LEE011) and SCH772984

We next determined the effects of the combination of trametinib or SCH772984 with LEE011 on regulation of the MEK/ERK signaling in NETs cells. Trametinib alone and in combination with LEE011 treatment significantly decreased pERK1/2 and cMyc expression in BON1, QGP-1, and NCI-H727 cells (Figure 5 and Figure S5).

Moreover, SCH772984 in combination with LEE011 synergistically upregulated the level of pERK1/2 in NCI-H727 but downregulated it in BON1 and QGP-1 cells (Figure 5 and Figure S5). SCH772984 plus LEE011 also synergistically upregulated levels of pMEK vs. any single agents in all three cell lines, indicating a potential loss of ERK1/2-mediated negative feedback inhibition in the RAS-RAF-MEK signaling in these cell lines. However, pERK1/2 expression was strongly inhibited after treatment with trametinib alone and almost completely eliminated after its combination with LEE011 in all three cell lines (Figure 5 and Figure S5).

Furthermore, trametinib alone or in combination with LEE011 upregulated pMEK1/2 levels in NCI-H727 and QGP-1 cells in a time-dependent manner (Figure 5 and Figure S5). The gradual increase in pMEK1/2 levels was more obvious at 72 h. Moreover, downstream pRSK levels showed modest downregulation in QGP-1 and NCI-H727 but not in BON1 after treatment with trametinib alone and in combination with LEE011 (Figure 5 and Figure S5). In contrast, SCH772984 alone or in combination with LEE011 caused an increase in pRSK levels in QGP-1 and NCI-H727, but there was no significant change in BON1. These data highlight the differential sensitivity of NETs to the MEK or ERK inhibitors and suggest that multiple signaling pathways other than the expected MEK/ERK activity might play roles in the inhibition of NET cell viability.

### 3.6. Synergistic Downregulation of the p38 and JNK Signaling after Treatment of NET Cells with Ribociclib (LEE011) and Trametinib or Ribociclib (LEE011) and SCH772984

**Figure 6:**
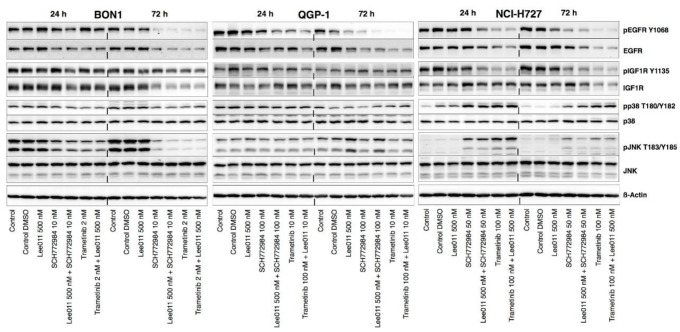
Effects of trametinib, SCH772984, and LEE011 in single treatments or combinations on the regulation of pEGFR, pIGFR, pJNK and pp38 signaling in NETs. NET cell lines (BON1, QGP-1, and NCI-H727) were grown and treated with trametinib, SCH772984, and LEE011 in single treatment or combinations for 72 h and assayed using Western blotting to assess levels of pEGFR, pIGFR, pp38, pJNK signaling proteins. Corresponding quantitative densitometry data are shown in Figure S6.

Treatment of NET cells with trametinib or SCH772984 was able to upregulate levels of pp38 and pJNK in NCI-H727, whereas it decreased them in BON1 and QGP-1 (Figure 6 and Figure S6). SCH772984 or trametinib in combination with LEE011 also dramatically enhanced the phosphorylation level of p38 and JNK vs. LEE011 alone in NCI-H727, whereas there was a synergistic tendency to decrease in BON1 (Figure 6 and Figure S6). Thus, it was concluded that the role of pJNK and pp38 signaling in the mediation of MEKi or ERKi was cell-context-dependent.

### 3.7. Variable Cell-Line-Dependent Effects of the MEKi Trametinib (TMT212), the ERKi SCH772984, and the CDK4/6i Ribociclib (LEE011) on PI3K/AKT Signaling

**Figure 7:**
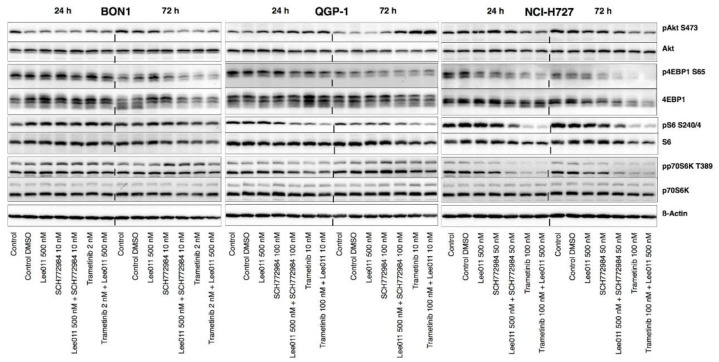
Effects of trametinib, SCH772984, and LEE011 in single treatments or combinations on the regulation of PI3K/AKT signaling in NETs. NET cell lines (BON1, QGP-1, and NCI-H727) were grown and treated with trametinib, SCH772984, and LEE011 in single treatments or combinations for 24 or 72 h, and assayed using Western blotting to assess levels of the PI3K/AKT signaling proteins. Corresponding quantitative densitometry data are shown in Figure S7.

Trametinib plus LEE011 was able to significantly reduce levels of pAKT in BON1 and NCI-H727 but not in QGP-1 as compared to each of the single agents, while reductions of p70S6K, pS6, and p4EBP1 were more pronounced in combination with LEE011 (Figure 7 and Figure S7). Moreover, the pAkt level was upregulated by SCH772984 in QGP-1 but decreased in BON1. In NCI-H727 cells, the level of pAkt was reduced at 24 h but rebounded back at 72 h after the SCH772984 treatment, alone or in combination with LEE011, although the expression of pS6 and p4EBP was decreased in all three cell lines and pp70S6K was reduced at 24 h and rebounded to baseline at 72 h in QGP-1 (Figure 7 and Figure S7). Thus, we can conclude that inhibition of the Ras/MAPK pathway or co-targeting of the CDK4/6 signaling led to the re-direction of cell signaling through the PI3K pathway and suggest that inhibition of the MEK/ERK pathway feeds back onto the PI3K/Akt pathway in a cell-line-dependent manner.

### 3.8. Regulation of the pEGFR and pIGFR Signaling after Combined Treatment of NET Cells with LEE011 and Trametinib or SCH772984

Trametinib or SCH772984 in combination with LEE011 caused significant reductions of pEGFR/EGFR levels vs. the controls (Figure 6 and Figure S6).

The levels of pIGFR after SCH772984 or trametinib treatment did not show any significant changes, although there was a decrease in BON1 cells after treatment with SCH772984 or trametinib plus LEE011 (Figure 6). Interestingly, SCH772984 reduced pIGFR levels in QGP-1 and NCI-H727 to a much lesser extent than trametinib. Trametinib or SCH772984 alone or their combination with LEE011 reduced pIGFR levels after a 24 h treatment; however pIGFR expression rebounded back at 72 h (Figure 6 and Figure S6).

## 4. Discussion

In the current study, we assessed the antitumor activities of the MEKi trametinib (TMT212) and the ERKi SCH772984 and evaluated their effects in combination with the CDK4/6i ribociclib (LEE011) in three different human NET cell lines in vitro and explored the underlying molecular events. We demonstrated that trametinib or SCH772984 in combination with LEE011 dose- and time-dependently inhibited NET cell growth (Figure 1 and Figure 2, Figures S1 and S2) and arrested tumor cells at the G1 phase of the cell cycle (Figure 3A–C), while trametinib alone and in combination with LEE011 also induced accumulation of cells in sub G1 events (Figure 3D), and induced a modest increase in NET cell apoptosis (Figure 4, Figures S2 and S4C). Molecularly, trametinib or SCH772984 in combination with LEE011 reduced levels of p-Rb, cyclinD1/cyclinD3, p-ERK, and c-myc, but upregulated pMEK, indicating that prevention of the negative feedback was activated by LEE011 alone. Moreover, their combination also inhibited phosphorylation of Akt, 4EBP1, p70S6K EGFR, IGFR, JNK p38, and others. The antitumoral effects were most prominent when NET cells were incubated with trametinib in combination with LEE011. Our findings are in accordance with the synergistic antitumoral effects of MEKi and CDK4/6i shown in various other tumor cell models, including neuroblastoma [23], neuroendocrine prostate carcinoma [43], melanoma [28,44,45], colorectal cancer [24,25,46], pancreatic cancer [47] and NSCLC models [26]. Our data suggest that it is worth further evaluating this combination of MEKi and CDK4/6i as an antitumoral strategy to control NETs in vivo. Serum concentrations of trametinib after a standard treatment dosage of 2 mg p.o. in humans are Cmax 22.2 ng/mL (CV 28%) [48], corresponding to approximately 0.04 uM. Serum concentrations of ribociclib after a standard treatment dosage of 600 mg p.o. in humans are Cmax 1680 ng/mL (CV 41%) [49], corresponding to approximately 3.9 uM. Thus, the concentrations we used in our in vitro experiments are pharmacologically relevant and can also be reached in vivo.

NET somatic mutations in *HRAS*, *KRAS*, *NRAS*, and *BRAF* are rare events that have been reported only in 1%, 8%, 0.7%, and 1% of patients, respectively [34]. In contrast, in NECs, somatic mutations of *KRAS* and *BRAF* are frequent events, which have been reported in 8% to 60% and 9% to 60% of tumors, respectively [50,51]. Treatment of *BRAF*-mutated NECs with the BRAF inhibitor dabrafenib and the MEK inhibitor trametinib prevented tumor growth in a xenograft model [52] and also have been reported in single-case reports of patients with metastatic NECs to cause an objective tumor response [51].

Our current in vitro study does have some limitations; for example, a previous study reported that the most efficient antitumor effects of MEKi and ERKi occurred on tumors harboring *BRAF* or *RAS* mutations, but our current study did not analyze such an association. However, the human pancreatic neuroendocrine tumor cell line BON1 harbors a mutation in *NRAS*, while the human pancreatic neuroendocrine tumor cell line QGP-1 harbors a mutation in *KRAS* [38]. Thus, our cell lines might be an appropriate model for alterations in the Ras/RAF/MEK/ERK signaling cascade.

Dysregulation of the CDK4/6-Rb pathway, as with the activation of CCND1/CDK4 or CDKN2A loss, contributes to cancer development and confers resistance of cancer cells to treatment with the MAPK/ERK inhibitors [53]. Conversely, CDK4/6i-resistant cells were sensitive to MEKi, indicating cancer cell dependence on the MAPK signaling in growth and invasion. Moreover, mutations of *RAS*, *BRAF*, *MEK1*, and *MEK2* could cooperate with *CDKN2A* loss in different cancers [54,55]. Thus, such alterations could lead to the combination of CDK4/6i with BRAFi or MEKi in preclinical models [47]. Indeed, clinical trials have been set to assess the use of CDK4/6i in combination with MEKi or ERKi to treat various solid tumors (NCT03170206, NCT02065063, NCT02857270, NCT02703571, and NCT03454035). Our current data are the first to show the antitumoral activity of the combination of CDK4/6i with MEKi or ERKi in NETs in vitro.

Furthermore, our current study revealed that trametinib and SCH772984 caused potent inhibitory effects on NET cells; both increased levels of pMEK in these three NET cell lines, while trametinib or SCH772984 in combination with LEE011 also upregulated the expression of pMEK. In contrast, trametinib alone or in combination with LEE011 induced durable suppression of pERK1/2 level. Moreover, SCH772984 alone or in combination with LEE011 reversed the upregulation of pERK by LEE011 alone. Although the inhibitory activity of trametinib and SCH772984 was not identical, trametinib seemed more potent in the suppression of MAPK and CDK4/6 than SCH772984. This phenomenon may be due to the inhibition of feedback mechanisms involving Sprouty and dual-specificity phosphatases [56]. Moreover, SCH772984 alone or in combination with LEE011 caused a pathway rebound in NCI-H727 cells, which may be because of the ERK feedback relief, as shown in the maintenance of pERK, pMEK, and p90RSK, but this was also possibly due to the low sensitivity of NCI-H727 to this agent or the combination. Meanwhile, the response of NET cells to SCH772984 may also be attenuated by many parallel pathways that potentially harbor the redundant regulation of the same downstream effectors by ERK1/2 or the reactivation of the ERK1/2 signaling in a RAF- or RAS-independent manner. Thus, it must be certain cell-line-specific feedback loops and sensitivities to these combination treatments to target the CDK 4/6/Rb and MAPK/ERK pathways and cause antitumor effects in vitro.

Again, the PI3K/Akt pathway is crucial in promoting cyclinD/CDK4/6-induced cell proliferation via regulation of the cell cycle progression [57]. In our current study, we observed that the PI3K/Akt compensatory activation in response to LEE011 was reversed after the combination of LEE011 with trametinib or SCH77284 in a cell-line-dependent manner, as shown by the decreases in pAKT, pS6K, and p4EBP levels after the combination treatment of LEE011 with SCH772984 or trametinib; however, the existence of tumor heterogeneity might explain some of the discordant results.

A previous study reported that trametinib blocked p38 MAPK phosphorylation, which was mainly due to a unique off-target effect of trametinib [58]. However, in our current study, we found that inhibition of pp38 after treatment with single agents or their combinations was inconsistent among these three NET cell lines (Figure 6 and Figure S6).

EGFR expression has been reported in 80–100% of NETs [34]. A previous study showed that EGF upregulation could mediate the acquired resistance to the CDK4/6 inhibitor [43]. In our current study, we found that trametinib or SCH772984 in combination with LEE011 significantly decreased pEGFR/EGFR levels (Figure 6 and Figure S6), indicating that the MEKi/ERKi was able to enhance the effects of LEE011 by suppressing the EGFR signal in NET cells.

In conclusion, our current study revealed potent antiproliferative effects of the MEKi trametinib in NETs. MEKi might be especially interesting as an antitumoral strategy in NECs, as NECs harbor a high rate of mutations in the *RAS/BRAF* signaling cascade [50,51]. In addition, the MEKi trametinib (TMT212) in combination with the CDK4/6i ribociclib (LEE011) showed synergistic antitumor activity in NET cell lines in vitro. Our findings are in accordance with synergistic antitumoral effects of the MEKi and CDK4/6i in various other tumor cell models, including neuroblastoma [23], neuroendocrine prostate carcinoma [43], melanoma [28,44,45], colorectal cancer [24,25,46], pancreatic cancer [47], and NSCLC models [26].

A major limitation of our in vitro study is the fact that the human neuroendocrine tumor cell lines BON1, QGP-1, and NCI-H727 are distinct from neuroendocrine tumors in patients regarding genome, tumor biology, and growth rate factors [37,38,39,59]. Patient-derived primary tumor cultures, spheroids, or organoids [60,61,62] or novel well-differentiated neuroendocrine tumor cell lines [63] might be more appropriate tumor models. The use of monolayer experiments in vitro is another limitation of our study. Further studies should be extended to 3D spheroid models [60,61,64,65], organoids [62], and in vivo animal models [66,67,68].

Clinical trials with MEKi in combination with CDK4/6i are ongoing in other solid tumors. The current preclinical results in neuroendocrine tumors still need to be evaluated in future preclinical and clinical studies. Future investigations in patient-derived primary tumor cultures, spheroids, and organoids [60,61,62] should also focus on somatic mutations or signaling cascade alterations to characterize predictive biomarkers in neuroendocrine tumors for the therapeutic response to dual MEK inhibition and CDK4/6 inhibition [19,69]. Clinical studies should also try to identify predictive genetic markers or biomarkers to optimize patient selection for this potentially individualized targeted therapy.

## 5. Conclusions

Inhibition of the MAPK/ERK signaling cascade by MEK inhibitors and ERK inhibitors in human neuroendocrine tumor cell lines in vitro demonstrates potent antiproliferative effects. Combination of the MEK inhibitor trametinib and the CDK4/6 inibitor ribociclib demonstrated synergistic antiproliferative effects in vitro. Further preclinical in vivo studies and clinical studies should investigate the therapeutic efficacy of MEK inhibitors alone and in combination with CDK4/6 inhibitors in neuroendocrine neoplasms.

## Data Availability

Data is contained within the article or supplementary material.

## Supplementary Materials

The following are available online at https://www.mdpi.com/2072-6694/13/6/1485/s1, Figure S1: Effects of ribociclib (LEE011), trametinib or SCH772984 on reduction of NET cell viability, Figure S2: Effects of trametinib, SCH772984, and LEE011 in single treatments or combinations on inhibition of NET cell viability, Figure S3: Effects of trametinib, SCH772984, and LEE011 in single treatments or combinations on apoptosis markes in NET cells, Figure S4: (A–C). Representative quantification results of western blots of LEE011 plus SCH772984 or Trametinib on CDK4/6-Rb signaling, apoptosis and cell cycle-related protein in NET cells (related to Figure 4), Figure S5: Representative quantification results of western blots of LEE011 plus SCH772984 or Trametinib on MEK/ERK signaling in NET cells (related to Figure 5), Figure S6: Representative quantification results of western blots of LEE011 plus SCH772984 or Trametinib on pEGFR, pIGFR signaling, pJNK and pp38 signaling in NET cells (related to Figure 6), Figure S7: Representative quantification results of western blots of LEE011 plus SCH772984 or Trametinib on PI3K/AKT signaling in NET cells (related to Figure 7).
